# Endoscopic Lateral Neck Dissection: A New Frontier in Endoscopic Thyroid Surgery

**DOI:** 10.3389/fendo.2021.796984

**Published:** 2021-12-22

**Authors:** Zeyu Zhang, Botao Sun, Hui Ouyang, Rong Cong, Fada Xia, Xinying Li

**Affiliations:** Department of Thyroid Surgery, Xiangya Hospital, Central South University, Changsha, China

**Keywords:** endoscopic thyroidectomy, robotic thyroidectomy, lateral neck dissection, quality of life, cosmetic outcome

## Abstract

**Background:**

Endoscopic thyroidectomy and robotic thyroidectomy are effective and safe surgical options for thyroid surgery, with excellent cosmetic outcomes. However, in regard to lateral neck dissection (LND), much effort is required to alleviate cervical disfigurement derived from a long incision. Technologic innovations have allowed for endoscopic LND, without the need for extended cervical incisions and providing access to remote sites, including axillary, chest–breast, face-lift, transoral, and hybrid approaches.

**Methods:**

A comprehensive review of published literature was performed using the search terms “lateral neck dissection”, “thyroid”, and “endoscopy OR endoscopic OR endoscope OR robotic” in PubMed.

**Results:**

This review provides an overview of the current knowledge regarding endoscopic LND, and it specifically addresses the following points: 1) the surgical procedure, 2) the indications and contraindications, 3) the complications and surgical outcomes, and 4) the technical advantages and limitations. Robotic LND, totally endoscopic LND, and endoscope-assisted LND are separately discussed.

**Conclusions:**

Endoscopic LND is a feasible and safe technique in terms of complete resection of the selected neck levels, complications, and cosmetic outcomes. However, it is recommended to strictly select criteria when expanding the population of eligible patients. A formal indication for endoscopic LND has not yet been established. Thus, a well-designed, multicenter study with a large cohort is necessary to confirm the feasibility, long-term outcomes, oncological safety, and influence of endoscopic LND on patient quality of life (QoL).

## 1 Background

In the past 10 years, the incidence of thyroid cancer (TC) has risen significantly among many districts and countries (1). Among the various subtypes, differentiated TC (DTC) accounts for approximately 95% of all TCs, and surgery is widely accepted as the most effective treatment for most DTC patients (2, 3). To date, conventional open thyroidectomy through a collar incision is the standard approach to thyroidectomy (4). While the majority of patients accept a thyroidectomy scar, a certain percentage of patients are not satisfied with a visible scar on the neck. Some of them with cosmetic needs may even seek out further plastic surgery to improve the appearance of the scar (5, 6). With the growing appreciation of cosmetic needs, as well as the progression of endoscopic technologies, many thyroidectomy approaches have undergone significant development and modification to minimize potential scars, including minimally invasive cervical approaches and remote-access approaches (7).

Neck appearance has an important influence on the quality of life (QoL) of TC patients. Endoscopic thyroidectomy and robotic thyroidectomy have been shown to be effective and safe surgical options for thyroid surgery while providing excellent cosmetic results (8, 9). For patients who need lateral neck dissection (LND) due to metastatic lateral neck lymph nodes (LNs), a conspicuous scar (L-shaped, U-shaped, or extended collar) is often created, causing unsatisfactory experiences after the surgery. In fact, much effort has been made to alleviate cervical disfigurement derived from long incisions (10). Endoscopic LND has limited acceptance for TCs with local metastasis, and only strictly selected patients are eligible for the technique. Technologic innovations have enabled LND without extending the cervical incision and allowing access to remote sites, including axillary, chest–breast, face-lift, transoral, and hybrid approaches. These techniques can also be divided into robotic approaches and endoscopic approaches, depending on the types of instruments used. The endoscopic approaches can be subdivided into total endoscopic and endoscope-assisted approaches by the way the devices are used.

In this review, we discussed the current knowledge regarding endoscopic LND. We specifically addressed the following points: 1) the surgical procedure, 2) the indications and contraindications, 3) the complications and surgical outcomes, and 4) the technical advantages and limitations. LND achieved through a robotic surgery system is recognized as robotic LND. Although there is an ambiguous border between total endoscopic LND and endoscope-assisted LND, we attempted to distinguish them in this review. Total endoscopic LND is performed with trocars in an enclosed area with or without CO_2_ insufflation, while endoscope-assisted LND is performed in a semiopen area without a trocar or CO_2_ insufflation.

## 2 Robotic Lateral Neck Dissection

### 2.1 Surgical Procedures

#### 2.1.1 Gasless, Transaxillary Approach

**Figure 1:**
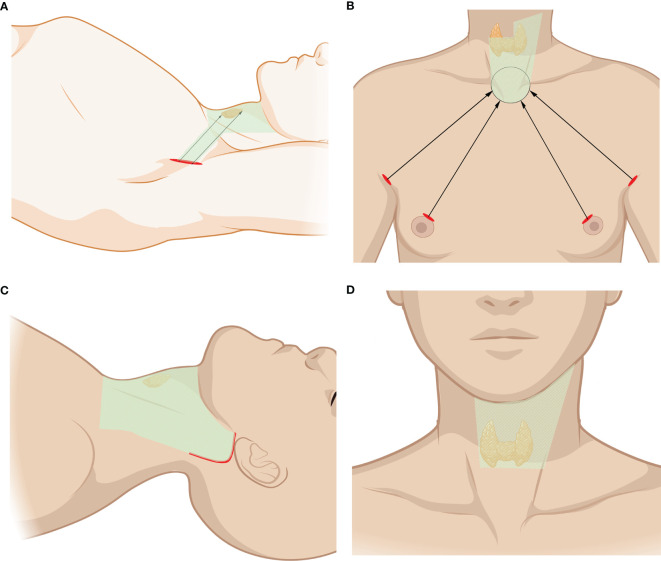
Robotic lateral neck dissection (LND) approaches. **(A)** Transaxillary approach. **(B)** Bilateral axillary breast approach (BABA). **(C)** Unilateral retroauricular approach (RA). **(D)** Transoral approach (TO); incision sites were the same in totally endoscopic TO approach. The area of raised skin flap was marked with green wireframes.

The robotic transaxillary approach is the most commonly reported robotic LND technique in the literature. A transaxillary approach was attempted *via* a 7- to 8-cm vertical incision located in the anterior axillary fold, and then a flap was created above the anterior surface of the pectoralis major muscle from the axilla to the clavicle (**Figure 1A**). The dissection should be continuously made until the submandibular gland and the posterior belly of the digastric muscle are reached. The skin flap is extended at the subplatysmal level in the neck superiorly to the mandible, posteriorly to the anterior border of the trapezius muscle, and anteriorly to the midline of the neck. The omohyoid muscle is interrupted at the thyroid cartilage, with the identification and dissociation of the internal jugular vein (IJV). A long and wide retractor blade is placed through the tunnel, and the flap and sternocleidomastoid muscle (SCM) are lifted to create a space for the operation. All four robotic arms are inserted through the same tunnel, and thyroidectomy and central neck dissection (CND) are performed as in conventional procedures. After the IJV is skeletonized and level III, IV, and VB dissections are performed, re-docking may be necessary for additional level II dissections. In addition, LNs located in levels IIB and VA, which are not routinely included in lymphadenectomy, can also be exposed and dissected (11–21).

#### 2.1.2 Bilateral Axillary Breast Approach

This approach requires four incisions, including a circumareolar incision in each breast and a vertical incision in each axilla. Compared with a simple thyroidectomy, a larger flap, up to the inferior border of the submandibular gland, superiorly to the angle of the mandible and posteriorly to the anterior edge of the trapezius, is needed for LND (**Figure 1B**). Two suture lines are used to pull the SCM laterally to expose the lateral cervical compartment, which can also be achieved by separating the SCM and the strap muscles. Similar to TA, the camera may need to be moved for better level II dissection. It is very important to preserve the brachial plexus and spinal accessory nerve (SAN) while dissecting level V LNs. Clipped level V LNs are moved to level IV through the bottom of the SCM. Additionally, to preserve the SAN, the posterior belly of the digastric muscle should be exposed while dissecting (22–25).

#### 2.1.3 Unilateral Retroauricular Approach

The retroauricular approach (RA) needs an L-shaped incision behind the auricle, and a flap is created above the SCM, proceeding anteriorly to the midline of the neck and extending superiorly to the submandibular gland and inferiorly to the level of the clavicle and sternal notch (**Figure 1C**). The flap is subsequently retracted and maintained by self-retaining retractors, and three arms of the da Vinci Surgical System are usually used in this approach. Specifically, the dissections are usually performed with the assistance of the robotic arms, while the exposure and dissection of the accessory nerve and LNs of levels II and III can be achieved under direct vision with regular surgical instruments (26, 27).

Kim and Byeon et al. reported a modified transaxillary and retroauricular (TARA) robot-assisted neck dissection. SAN can be identified through the RA incision. LNs of level II, VA, and upper level III can be recognized and dissected under direct vision, while the LNs of levels III, VB, and IV can be recognized and dissected with the assistance of robotic arms through the transaxillary approach. Thyroidectomy and CND can also be accomplished *via* TA (28, 29).

#### 2.1.4 Transoral Approach

Few studies have applied TO during robotic dissection. Tae et al. reported one case of dissecting LNs of levels III and IV. One central incision and two lateral incisions were made on the base of the lower lip frenulum and the oral commissure. A flap is required inferiorly to the sternal and clavicle notch and laterally to the SCM. Another incision is required in the right axillary region for a third robotic instrument (**Figure 1D**). Total thyroidectomy and bilateral CND are similar to those of the endoscopic transoral approach. The SCM can be retracted to obtain a better view of the operation. With good exposure and protection of the IJV, the LNs of levels III and IV can be subsequently dissected (30).

### 2.2 Indications and Contraindications

#### 2.2.1 Gasless, Transaxillary Approach

Based on the experience of centers that have performed this procedure thus far, the inclusion criteria are as follows: 1) unilateral DTC with cervical LN metastasis and 2) without previous surgical treatments of TC. The exclusion criteria are usually as follows: 1) unrelated pathologic conditions of the neck or shoulder; 2) extensive extrathyroidal spread; 3) metastatic LNs are fused or fixed; 4) metastatic LNs on the substernal or the infraclavicular area; 5) recurrent disease; and 6) distant metastasis.

#### 2.2.2 Bilateral Axillary Breast Approach

The inclusion criteria are as follows: 1) primary tumor size <3 cm without local invasion; 2) LN metastasis derived from DTC; 3) largest LN diameter <2 cm; and 4) an advanced cosmetic need. The exclusion criteria are usually as follows: 1) previous neck or chest surgery; 2) body mass index (BMI) >30 or short neck; 3) extensive extrathyroidal spread; 4) metastatic LNs in the level V regions or below the sternoclavicular joint; 5) metastatic LNs fused or fixed; and 6) distant metastasis.

#### 2.2.3 Unilateral Retroauricular Approach

The inclusion criteria for RA are as follows: 1) unilateral tumor and 2) with no previous history of treatment for thyroid carcinoma. The exclusion criteria are as follows: 1) recurrent disease; 2) unrelated pathologic conditions of the retroauricular area; 3) extensive extrathyroidal spread; 4) metastatic LNs fused or fixed; and 5) distant metastasis.

#### 2.2.4 Transoral Approach

The inclusion criteria for the transoral approach are as follows: 1) primary tumor size <2 cm in the unilateral lobe; 2) without capsular invasion; 3) suspected level III/IV LN metastases; 4) an advanced cosmetic need; and 5) no evidence of thyroiditis. The exclusion criteria are as follows: 1) previous neck or chest surgery; 2) BMI > 30 or short neck; 3) extensive extrathyroidal spread; 4) metastatic LNs in the level I, II, or V regions; 5) level III or IV metastatic LNs fused or fixed; and 6) distant metastasis.

### 2.3 Complications and Outcomes

He et al. reported a cohort of 260 patients treated *via* the bilateral axillary breast approach (BABA). After surgery, 19.6% of patients developed transient hypocalcemia, and 1.15% developed transient hoarseness without permanent hypocalcemia or hoarseness. A total of 1.15% of patients developed a seroma, while less than 1% of patients have less chylar leakage or tracheal fistula (24). In Kim’s cohort, they reported 42 patients undergoing robotic modified radical neck dissection through TA with a 5-year follow-up time, and a 1:3 matching analysis with the conventional open procedure was conducted. Their results showed no significant difference in the complication rate between groups (58.5% in the robotic group and 62.7% in the conventional group), with transient hypocalcemia as the most common complication in both groups. Lymphatic fistula in one case and transient hypocalcemia in three (6.2%) cases were reported in Lira’s cohort with an RA. No other surgical complications were described (26). In other comparative studies, the reported complication rates were not different between robotic and open surgeries (12, 14, 20, 23, 26). Minor chyle leakages, temporary recurrent laryngeal nerve (RLN) palsy, arm movement disorders, and Horner syndrome have been noticed after robotic surgeries (12, 20). Kim et al. reported as many as 500 cases of robotic LND. Permanent hypocalcemia, permanent RLN injury, Horner’s syndrome, vagus nerve injury, and wound infection were noticed with extremely low rates (21). The general characteristics, technical safety (complications), and oncological outcomes of robotic surgeries are listed in **Table 1**.

**Table 1:** General characteristics, technical safety, and oncological outcomes of robotic studies.

Surgeons	Instruments/approaches	No. of cases	Study type	Dissected neck levels	Complications (n)	Difference in retrieved nodes*	Follow-up time (months) and recurrence (n)	Ref
Kim et al.	Robotic transaxillary^#^	42	Retrospective/comparative	IIA, IIB, III, IV, and Vb	Seroma, 4 Chyle leakage, 4 Temporary vocal cord paresis, 3 Transient hypocalcemia, 18 Arm movement disorder, 1	No difference	5 years 1	(12)
Lira et al.	Robotic retroauricular	12	Retrospective/case series	II–V	Reoperation, 1 Transient hypocalcemia, 2 Vocal cord paresis, 3 Surgical site infection, 1	NA	17.4 (mean) 0	(26)
Tae et al.	Robotic transoral	1	Case report	III, IV	None	NA	NA NA	(30)
Yu et al.	Robotic BABA	15	Retrospective/case series	II–V	Transient hypocalcemia, 7 Temporary vocal cord paresis, 1 Horner’s syndrome, 1	NA	18.7 (mean) 0	(22)
Byeon et al.	Robotic retroauricular	4	Retrospective/case series	II–V	Seroma, 1 Transient hypocalcemia, 2 Chyle leakage, 1	NA	NA NA	(27)
Kim et al.	Robotic BABA	13	Retrospective/comparative	II, III, IV, and Vb	Chyle leakage, 1	No difference	15.9 (mean) 0	(23)
He et al.	Robotic BABA	260	Retrospective/case series	II, III, IV, and Vb**	Transient hypocalcemia, 51 Temporary vocal cord paresis, 3 Seroma, 3 Surgical site infection, 1 Tracheal fistula, 1	Presenting as number of retrieved nodes	28.6 (mean) 0	(24)
Byeon et al.	Robotic TARA	1	Case report	III, IV	None	Presenting as number of retrieved nodes	NA NA	(29)
Kim et al.	Robotic TARA	22	Retrospective/comparative	II–V	Seroma/hematoma, 3 Transient hypocalcemia, 6 Chyle leakage, 1 Earlobe numbness, 6 Temporary vocal cord paresis, 2	No difference	15.9 (mean) 0	(28)
Song et al.	Robotic BABA	4	Retrospective/case series	Bilateral MRND***	Pleural effusion (not chylous)	Presenting as number of retrieved nodes	17-36 (range) 0	(25)
Kang et al.	Robotic transaxillary	56	Retrospective/comparative	IIA, III, IV, and Vb	Seroma/hematoma, 5 Transient hypocalcemia, 27 Chyle leakage, 5 Temporary vocal cord paresis, 2 Reoperation, 1	No difference	One year (fixed time point) 0	(20)
Lee et al.	Robotic transaxillary	62	Retrospective/comparative	IIA, III, IV, and Vb	Seroma/hematoma, 2 Transient hypocalcemia, 24 Chyle leakage, 1 Temporary vocal cord paresis, 2	No difference	8.4 (mean) 0	(14)
Kim et al.	Robotic transaxillary	500	Retrospective/case series	II–V	Transient hypocalcemia, 151 Permanent hypocalcemia, 20 Temporary vocal cord paresis, 20 Permanent vocal cord paresis, 5 Seroma/hematoma, 19 Chyle leakage, 26 Horner’s syndrome, 2 Vagus nerve injury, 1 Wound infection, 1	Presenting as number of retrieved nodes	NA 5	(21)

NA, not available; MRND, modified radical neck dissection; LND, lateral neck dissection.

^#^Only studies regarding robotic transaxillary approaches with case number >40 have been included.

^*^Compared with those retrieved in conventional open surgery.

^**^Unilateral LND was performed in 239 cases and bilateral LND in 21 cases.

^***^Dissected levels were not described.

In He’s cohort of BABA procedures, all 260 patients experienced varying degrees of paraesthesia in the neck and chest; however, they mainly recovered within 4–12 months. All of the patients were extremely satisfied or satisfied with the cosmetic results. Only one case was identified with a tumor recurrence in the short-term follow-up period (1–48 months) (24). Lee’s comparative study concerning TA and conventional thyroidectomy investigated pain scores, paraesthesia, and cosmetic satisfaction. While no significant difference was detected in pain scores, paraesthesia of the neck was found more frequently after conventional thyroidectomy; however, paraesthesia of the chest was more frequent in the robotic group. Moreover, the robotic group had better cosmetic satisfaction than the conventional group, and the neck and shoulder function scores were not different between the two groups (14). The cosmetic effects and other QoL results are listed in **Table 2**.

**Table 2:** Cosmetic results and other quality of life (QoL) results in retrieved references.

Surgeons	Cosmetic evaluation methods	Cosmetic results	Other QoL evaluation	Other results	Ref
He et al.	Five-point scale	4.68 ± 0.35 (score)*	None	None	(24)
Kim et al.	Five-point scale	3.9 ± 1.0, better than open group	None	None	(28)
Lee et al.	Five-point scale	Better than open group**	Pain score, voice handicap index, etc.^#^	No difference	(14)
Song et al.	Five-point scale	1.64 ± 0.61 (one month), better than open group	Pain and paresthesia scores of the neck and anterior chest area	No difference or higher in robotic group***	(13)
Guo et al.	NA	8.3 ± 0.7, better than open group	Pain scores	No difference	(31)
Lin et al.	Visual analog scale (VAS)	9 (mean, range 5–10)*	Pain score, voice handicap index, etc.^#^	Presenting as scores	(32)
Zhang et al.	Verbal response scale and numeric rating scale	2.8 ± 0.5 and 7.0 ± 0.9, better than open group	Pain score (VAS)	Better in endoscopic assisted group	(33)
Zhang et al.	Verbal response scale and numeric rating scale	7.0 ± 1.2 and 2.7 ± 0.6, Better than open group	None	None	(34)
Lin et al.	Verbal response scale	9 (mean, range 9–10), better than open group	Pain score, voice handicap index, etc.^#^	No difference	(35)
Zhang et al.	Five-point scale	1.4 ± 0.6 (3 months), better than open group	Pain and paresthesia scores of the neck	Better in endoscopic assisted group	(36)

NA, not available.

^*^Case series studies.

^**^Presenting as numbers of satisfied or dissatisfied patients.

^***^Pain and paresthesia scores of the neck were similar as those in open surgery, while pain and paresthesia scores of the anterior chest area were higher.

^#^Other QoL evaluation included swallowing impairment score (SIS-6), neck dissection impairment index (NDII), and arm abduction test (AAT) in these studies.

Meanwhile, robotic surgeries are as effective in harvesting LNs as conventional surgery (20). Serum triglyceride (TG) levels (thyroid-stimulating hormone (TSH) suppressed), neck ultrasound, ultrasound-guided fine-needle aspiration (FNA), CT, and PET-CT were used to evaluate disease recurrence. Multiple studies have revealed that the recurrence and mortality rates are not different between robotic and open surgeries (13, 16, 20, 23, 24). In Kim’s two cohorts investigating cancer recurrence, recurrence rates over 5 years (the longest follow-up period as reported) did not differ between the robotic and open groups (2.4 vs. 2.9%), showing a consistent oncological effect (12, 21).

### 2.4 Technical Advantages and Limitations

Robotic instruments have shown additional superiorities, over other endoscopic instruments, including tremor-free, stabilized, and 7-degree freedom movement of instruments; 3-dimensional endoscopic view; self-controlled traction; and optimized ergonomics. Delicate anatomical operation with versatile instruments can assist with complex intraoperative situations and lead to easier preservation of the parathyroid gland and the identification of nerves, vessels, and lymphangion. With good exposure, dissection of all levels of the neck compartments can be theoretically achievable. In Kim’s experience, setting contraindications of robotic LND according to the TNM staging system is unnecessary. They believe that experienced surgeons can determine whether they can complete robotic LND even though a patient may have metastatic nodes in level VII or primary cancer can be a T4 lesion (21). This suggests that the indication for robotic LND can be easily further expanded with growing experience. Several technical difficulties and limitations in the application of robot-assisted LND are as follows: 1) a longer operative time; 2) a wider flap dissection is needed, which means greater trauma and more severe paraesthesia of the flap area; 3) higher costs; 4) a prominent clavicle may make it hard to completely remove LNs of level IV using the TA and BABA approaches; 5) the upper neck (level II) is difficult to remove (except in the RA approach); and 6) splitting the SCM may exaggerate neck pain and stiffness, especially with bilateral procedures.

## 3 Totally Endoscopic Lateral Neck Dissection

### 3.1 Surgical Procedures

#### 3.1.1 Chest–Breast Approach

**Figure 2:**
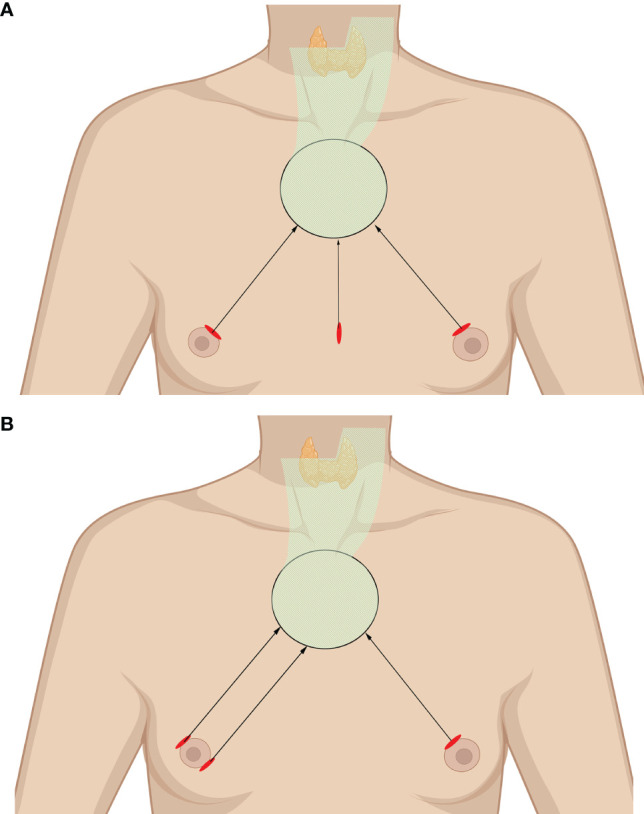
Totally endoscopic lateral neck dissection (LND) approaches. **(A)** Chest–breast approach. **(B)** Total mammary areolas approach. The area of raised skin flap was marked with green wireframes.

The chest–breast approach is characterized by CO_2_ gas usage and the presence of all of the incisions and working space confined to the anterior chest wall, including the breast. Various total endoscopic approaches have been described, such as approaches *via* the parasternum and bilateral mammary areolas (chest–breast, **Figure 2A**) (37) and total mammary areolas (breast, **Figure 2B**) (38, 39). Epinephrine solution or liquid–gas mix tumescent is used to control bleeding while creating the flap. One 10-mm incision at the margin of the breast (parasternum) or at the right areola for the laparoscope and two 5-mm incisions at the bilateral areola for operating the instruments are made for the trocar location. The working space is filled with CO_2_, maintaining a pressure of approximately 7 mmHg. Devices have been introduced in this kind of approach, such as U-shaped retractors, suspension sutures, ultrasonic coagulators, and minilap (31, 37–43).

After thyroidectomy and CND, the working space should be expanded superiorly to the lower edge of the submandibular gland and laterally to the lateral edge of the SCM. The steps of LND are basically identical to those of conventional thyroidectomy after the SCM is split longitudinally to expose the lateral cervical compartment. Particularly, the posterior border of the IJV and the SAN should be carefully anatomized when removing the LNs of levels 2A and 2B. The transverse cervical artery, phrenic nerve, vagus nerve, brachial plexus, and cervical plexus are exposed and protected (31, 37–43).

#### 3.1.2 Transoral Approach

Tan et al. reported selective LND *via* a transoral endoscopic approach in 20 PTC cases. Ngo et al. also reported a case of LND *via* a transoral endoscopic approach. Three vestibular incisions were made in the mouth vestibular (**Figure 1D**). The working space is described as between the sternohyoid muscles and the platysma muscle. Upon completing the thyroid lobectomy and CND, selective LND is performed through the two heads of the SCM. After removal of the omohyoid muscle to create a larger space, the IJV should be anatomized and well protected. The LNs of levels III and IV can be subsequently resected starting from the level of the carotid bifurcation to the clavicle (44, 45).

### 3.2 Indications and Contraindications

#### 3.2.1 Chest–Breast Approach

Based on the experience of centers that have performed this procedure thus far, the inclusion criteria for chest–breast approach are as follows: 1) primary tumor size <3 cm without local invasion; 2) LN metastasis derived from DTC; 3) largest LN diameter <2 cm; and 4) an advanced cosmetic need. The exclusion criteria are usually as follows: 1) previous neck or chest surgery; 2) BMI >30 or short neck; 3) extensive extrathyroidal spread; 4) metastatic LNs in the level V regions or below the sternoclavicular joint; 5) metastatic LNs fused or fixed; and 6) distant metastasis (39, 40, 42).

#### 3.2.2 Transoral Approach

The inclusion criteria for the transoral approach are as follows: 1) primary tumor size <2 cm in the unilateral lobe; 2) without capsular invasion; 3) suspected level III/IV LN metastases; 4) an advanced cosmetic need; and 5) no evidence of thyroiditis. The exclusion criteria are as follows: 1) previous neck or chest surgery; 2) BMI > 30 or short neck; 3) extensive extrathyroidal spread; 4) metastatic LNs in the level I, II, or V regions; 5) level III or IV metastatic LNs fused or fixed; and 6) distant metastasis (44).

### 3.3 Complications and Outcomes

Several centers have reported the perioperative complications of totally endoscopic LND. Common complications of thyroidectomy and lymphadenectomy may also occur during endoscopic surgery. Yan et al. performed and reported their rich experience with endoscopic thyroidectomy *via* a breast approach with levels II, III, and IV LND. They retrospectively compared the endoscopic LND group with 155 patients and the conventional open LND group with 102 patients. Operation-related complications, such as transit hoarseness, hematoma, IJV rupture, and limb lift restriction occurred in both the endoscopic LND group and the open group. No significant difference was found for the complication incidences between the groups. However, the mean operating duration was longer in the endoscopic LND group than in the open group (40). Guo et al. also confirmed the prolonged LND time of endoscopic thyroidectomy. Consistently, no significant difference was found in transient voice change, transient hypoparathyroidism, postoperative lymphatic leakage, or intraoperative large blood vessel injury, while no severe complications, such as hematoma, permanent hypoparathyroidism, and permanent nerve injury were found to occur (31). Wang et al. reported one case of conversion to open surgery due to right RLN invasion. Injury to the SAN was found in 2 patients, who subsequently developed light SCM dystrophy (41). Cervical plexus and hypoglossal nerve injury have been reported in Chen’s study (46). The general characteristics, technical safety (complications), and oncological outcomes of totally endoscopic surgeries are listed in **Table 3**.

**Table 3:** General characteristics, technical safety, and oncological outcomes of totally endoscopic and endoscope-assisted studies.

Surgeons	Instruments/approaches	No. of cases	Study type	Dissected neck levels	Complications (n)	Difference in retrieved nodes*	Follow-up time (months) and Recurrence (n)	Ref
Guo et al.	Totally endoscopic/chest–breast^#^	24	Retrospective/case series and comparative	II, III, IV	Blood vessels injury, 2 Transient hypocalcemia, 4 Chyle leakage, 2 Temporary vocal cord paresis, 1	No difference	NA 0	(31, 47)**
Huo et al.	Totally endoscopic/chest–breast	20	Retrospective/case series	II, III, IV	Blood vessels injury, 2 Chyle leakage, 1 Temporary vocal cord paresis, 1 Transient hypocalcemia, 4	NA	NA NA	(43)
Wang et al.	Totally endoscopic/chest–breast	37	Retrospective/case series	II– V	Temporary vocal cord paresis, 3 Transient hypocalcemia, 12 Permanent hypocalcemia, 1 Spinal accessory nerve injury, 2 Chyle leakage, 1 Chest wall ecchymosis, 1	Presenting as number of retrieved nodes	24 (mean) 1	(41)
Wang et al.	Totally endoscopic/chest–breast	155	Retrospective/comparative	II, III, IV	Temporary vocal cord paresis, 8 Seroma, 3 Chyle leakage, 4 Infection, 2 Internal jugular vein rupture, 19 Limb lift restriction, 6	No difference	10 years (max) 2	(37, 40, 42)**
Chen et al	Totally endoscopic/chest–breast	35	Retrospective/case series	IIA, III, IV	Chyle leakage, 1 cervical plexus injury, 7 hypoglossal nerve injury, 1 accessory nerve injury, 3 internal jugular vein injuries, 2	Presenting as number of retrieved nodes	18.1 (mean) None	(46)
Huo et al.	Totally endoscopic/chest–breast	12	Retrospective/comparative	II, III, IV, VB	Chyle leakage, 1 Temporary vocal cord paresis, 2	No difference	NA None	(48)
Kitagawa et al.	Endoscope assisted/anterior chest	3	Retrospective/case series	Lateral zone***	None	NA	NA NA	(49)
Wu et al.	Endoscope assisted	26	Retrospective/case series	IIA, III, IV	Temporary vocal cord paresis, 2 Transient hypocalcemia, 4	Presenting as number of retrieved nodes	19 (mean) None	(10)
Zhang et al.	Endoscope assisted	26	Retrospective/case series	II– V	Spinal accessory nerve injury, 1	Presenting as number of retrieved nodes	NA NA	(50, 51)**
Zhang et al	Endoscope assisted	130	Retrospective/case series	II– IV or II– V	Transient hypocalcemia, 19 Temporary vocal cord paresis, 7 Permanent vocal cord paresis, 3 Spinal accessory nerve injury, 2 Chyle leakage, 4 Seroma, 1	Presenting as number of retrieved nodes	19 (mean) None	(34, 52)**
Lin et al.	Endoscope assisted	18	Retrospective/case series	II– IV	Transient hypocalcemia, 1	NA	54.5 (mean) None	(32)
Zhang et al.	Endoscope assisted	32	Prospective/comparative	II– IV, VB	Temporary vocal cord paresis, 1 transient hypocalcemia, 4 Seroma, 1 Horner’s syndrome, 1 Chyle leakage, 1	No difference	NA None	(33)
Lin et al.	Endoscope assisted/anterior chest	31	Retrospective/comparative	II– IV	Transient hypocalcemia, 2	No difference	NA None	(35)
Zhang et al	Endoscope assisted	18	Prospective/comparative	II– V	Temporary vocal cord paresis, 6 Transient hypocalcemia, 3	No difference	NA NA	(36)

NA, not available.

^#^Only studies regarding total totally endoscopic/chest–breast and endoscope assisted (collar incision) with case number >10 have been included.

^*^Compared with those retrieved in conventional open surgery.

^**^These studies may include repeatedly reported cases.

^***^Dissected levels were not described.

In contrasting analyses, no difference was found in the number of dissected lateral LNs between the endoscopic LND group and the conventional open LND group (31). Yan et al. calculated the number of dissected LNs in levels II, III, and IV. The numbers at each level were statistically identical in the endoscopic group and open group (40). Cosmetic satisfaction scores were mentioned in only one study, and the endoscopic group had a higher score than the open group (**Table 2**) (31). The median follow-up period in Wang’s cohort was 24 months, ranging from 4 to 59 months. Postoperative thyroglobulin (Tg) levels were used to evaluate tumor recurrence, and Tg < 1 ng/ml was achieved in 80% of individuals 1 year after surgery, with only one recurrence at the LNs of level VII. All 37 patients were satisfied with the cosmetic results (41). The study by Yan et al. reported local recurrence in four patients (2 in the endoscopic group and 2 in the open group) during follow-up, while no patients developed distant metastases or tumor-related death (40).

### 3.4 Technical Advantages and Limitations

Although the endoscopic device is not as versatile as the robotic device, it is easy and economical to apply in most hospitals. The effectiveness and safety of endoscopic surgery have been widely proven to be the same as those of robotic surgery in the treatment of TC. However, it is slightly inferior to robotic surgery in terms of the diversity of surgical approaches and the range of indications. Several technical difficulties and limitations in the application of endoscopic thyroidectomy and LND are as follows: 1) unexpected complications in endoscopic instrument usage, such as CO_2_ embolism, tumor or metastatic LN rupture, and chest wall ecchymosis. 2) Poor surgical techniques, working space building, and lateral compartment exposure lead to a longer surgical time, which means a longer anesthesia time, increased cost, and a higher incidence of possible complications. 3) At present, the cosmetic evaluations are only descriptive. A quantitative, reproducible scoring system is needed. 4) In the breast approaches, level IV and VI lymph dissections are restricted by claviculation, while level V lymph dissections are restricted by the lateral border of the SCM. Due to endoscopic magnification and less space restriction, it will be easier to perform level IIB LND with endoscopic techniques than with open surgery. 5) Difficulty in level II and V lymph dissection with oral approaches: it will be difficult to expose level II LNs during surgical manipulation from the superior to inferior direction. Level V LNs are also restricted by the lateral border of the SCM. 6) SCM stiffness: in all of the studies, the SCM was split longitudinally to expose the lateral cervical compartment. However, the consequences of SCM splitting, such as stiffness, numbness, pain, and constriction, have not been evaluated.

## 4 Endoscope-Assisted Lateral Neck Dissection

### 4.1 Surgical Procedure

**Figure 3:**
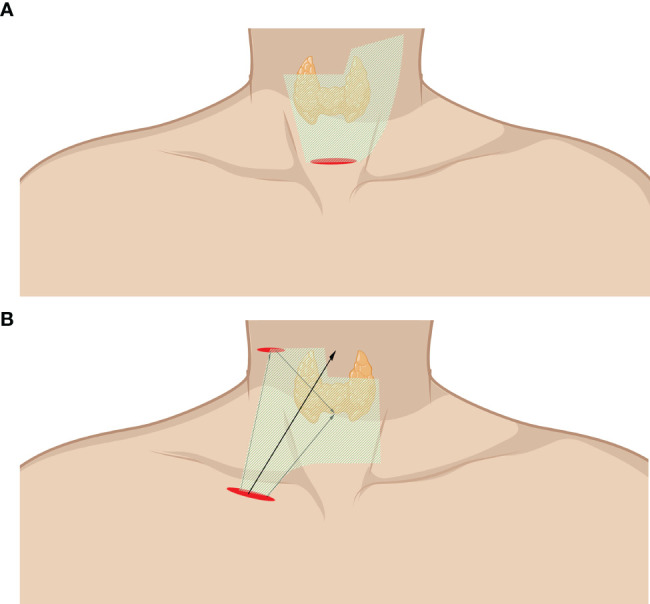
Endoscope-assisted LND approaches. **(A)** Approach through the collar incision. **(B)** Approach through chest wall incision (infraclavicular incision). The area of raised skin flap was marked with green wireframes.

Endoscope-assisted LND is characterized by the endoscopic removal of lateral LNs through an incision of approximately 2–4 cm without a trocar. Unlike total endoscopic LND, the working space of endoscope-assisted LND is not fully enclosed and is often remedied by a gasless traction system. Special instruments are introduced to lift the anterior neck skin without CO_2_ gas as originally described by Miccoli and Lombardi (53, 54). Zhang et al. invented a working space creator and reported a cohort with the largest number of cases by using this instrument (34, 52). The working space is created from the posterior belly of the digastric muscle to the posterior border of the SCM (**Figure 3A**). The SCM and IJV are dissected and retracted reversely to expose the lateral lymph. The vagus nerve is carefully anatomized, followed by the removal of the LNs and surrounding fibroadipose tissue. Level II, III, and IV LNs can be dissected in the caudad to cephalad direction, while level III and IV LNs can also be dissected in the cephalad to caudad direction due to freedom from restriction. The SAN, cervical plexus, phrenic nerve, brachial plexus, and transverse cervical should be carefully protected (10, 32–34, 50–53).

Kitagawa and Lin et al. reported a totally gasless anterior chest approach. A 3.5-cm incision and another 0.5-cm incision were made on the tumor side of the chest wall and the lateral neck with a widely created working space (**Figure 3B**). The incision on the lateral neck should be widened to 2 cm when LND is required. The working space is subsequently widened to the level of the lobulus auriculae, the posterior belly of the digastric muscle, the posterior border of the SCM, and the space beneath the SCM (35, 49).

### 4.2 Indications and Contraindication

The eligibility criteria were usually as follows: 1) unilateral tumor and 2) largest diameter <4 cm. The exclusion criteria were usually as follows: 1) extrathyroidal extension; 2) distant metastases; 3) LN metastases in level I or VA; and 4) history of previous neck surgery or radiation therapy (33, 34, 49).

### 4.3 Complications and Outcomes

Thyroidectomy and CND are performed as conventional open procedures. Transient RLN palsy and hypoparathyroidism rates are similar to those of the conventional open procedure. Wu et al. reported no conversion to open surgery, no significant blood loss, and only mild postoperative pain in their 26 cases (10). Zhang et al. reported an injury of the trapezius branch of the accessory nerve, causing weakness that recovered within 2 years (50). Kitagawa reported lateral LN clearance in three patients through a totally gasless anterior neck skin lifting method without any severe complications (49). Zhang et al. designed a prospective randomized study to compare video-assisted and open LND. One transient sympathetic nerve injury and one minor chyle leak were observed in the video-assisted group (33).

Cosmetic outcomes were generally better in endoscope-assisted surgery than in open surgery (32, 34, 36, 52). In Zhang’s cohort, postoperative pain was significantly less severe in the endoscope-assisted group at 24 and 48 h after surgery (33). The incidence of voice change, swallowing, neck impairment, and arm abduction was not significantly different in Lin’s cohort (the details can be found in **Table 2**) (35). In Wu’s cohort, the postoperative Tg level was dramatically decreased to zero in most patients. Eleven patients received ^131^I diagnostic whole-body scans at the 6- to 12-month follow-up, and no recurrence or metastasis was found (10). In Zhang’s prospective cohorts, no patients showed any uptake of ^131^I outside the thyroid bed as assessed by whole-body scans performed after the administration of 100 mCi of ^131^I. Neck ultrasound showed no tumor recurrence or residual disease in either group during follow-up (33).

### 4.4 Technical Advantages and Limitations

Unlike other remote extracervical approaches, endoscope-assisted LND is characterized by less trauma, a shorter learning curve, and less demand for specific instruments. When uncontrolled bleeding or unexpected aggressiveness of the disease occurs, it is also not hard to convert to conventional thyroidectomy. As it is similar to the conventional open procedure, the indications for endoscope-assisted LND can be easily extended with growing experience. The extension of lateral neck clearance has been reported to include levels IIA, IIB, III, IV, and partly V (33). Zhang et al. have made some efforts to extend the indications, showing the feasibility of endoscopic upper mediastinal LN dissection in the treatment of papillary thyroid carcinoma with promising results (55).

Several technical difficulties and limitations in the application of endoscope-assisted LND still exist: 1) it leaves an obvious scar on the neck, although it is better than the scar from conventional thyroidectomy. 2) LNs of level I and VA are hard to reach and remove. 3) It has a longer operative time (which will decline rapidly with greater experience).

## Conclusions and Future Perspectives

Endoscopic LND allows comprehensive en bloc removal of all lateral neck levels through a remote extracervical incision or an unextended collar incision. They are feasible and safe techniques in terms of the complete resection of selected neck levels, complications, and cosmetic outcomes. As the most commonly reported approach, robotic LND has more selective remote sites for robotic system docking. Device intelligence makes the theoretical indications less limited, but the obvious disadvantage is the high cost. In general, endoscopic LND is easy to carry out. The completeness of the surgical resection in selected compartments is satisfactory; however, some neck levels are difficult to achieve. Endoscope-assisted LND (cervical approach) is characterized by less trauma, close to open vision, and not limited by local tumor progression, and it can even be feasible for mediastinal LN dissection. Nevertheless, an obvious scar is still left on the neck. There is still controversy concerning the technical challenges, the introduction of new complications, and the operative trauma of endoscopic LND. Technical difficulties and limitations affect the different approaches. It is also challenging for all of the techniques to dissect all of the metastatic LNs in hard-to-reach areas or invaded areas. Moreover, unlike other procedures, additional trauma following the creation of the working space is inevitable due to the lack of free space in the neck. Theoretically, endoscopic approaches can provide meticulous dissection in the lateral neck. Complete surgical resection, a reduction in the time required, and less trauma will be achieved with the growing experience of surgeons. Regardless, it is recommended to strictly apply selection criteria when expanding the population of eligible patients in centers with a high volume of thyroid surgery. A formal indication for endoscopic LND has not yet been established. Thus, a well-designed, multicenter study with a large cohort is necessary to confirm the feasibility, long-term outcomes, oncological safety, and influence of endoscopic LND on patient QoL.

## Author Contributions

All authors contributed to drafting or revising the article, gave final approval of the version to be published, agreed to the submitted journal, and agree to be accountable for all aspects of the work.

## Funding

This work was supported by the National Natural Science Foundation of China (grant no. 82073262) and the Hunan Province Natural Science Foundation (grant no. 2019JJ40475).

## Conflict of Interest

The authors declare that the research was conducted in the absence of any commercial or financial relationships that could be construed as a potential conflict of interest.

## Publisher’s Note

All claims expressed in this article are solely those of the authors and do not necessarily represent those of their affiliated organizations, or those of the publisher, the editors and the reviewers. Any product that may be evaluated in this article, or claim that may be made by its manufacturer, is not guaranteed or endorsed by the publisher.
